# Cloning, Identification, and Functional Analysis of the *Foxl2* Gene in *Procambarus clarkii*

**DOI:** 10.3390/genes14122190

**Published:** 2023-12-08

**Authors:** Jin Huang, Weilin Zhu, Min Peng, Chunling Yang, Xiaohan Chen, Tiejun Wu, Digang Zeng, Yongzhen Zhao, Xiuli Chen

**Affiliations:** 1 Guangxi Key Laboratory of Aquatic Genetic Breeding and Healthy Aquaculture, Guangxi Academy of Fishery Science, Nanning 530021, China; 2118303002@st.gxu.edu.cn (J.H.); gxnnpm@126.com (M.P.); scsycl@163.com (C.Y.); chenxhn@163.com (X.C.); wtj5111@163.com (T.W.); swmisys@126.com (D.Z.); fisher1152002@126.com (Y.Z.); 2 College of Animal Science and Technology, Guangxi University, Nanning 530005, China

**Keywords:** *Procambarus clarkii*, *Foxl2*, clonal expression, in situ hybridization, RNAi

## Abstract

*Procambarus clarkii* is the most widely distributed freshwater shrimp in China, with important economic value and great potential for development. The forkheadboxL2 (*Foxl2*) gene has been found to be involved in the reproductive development of many crustaceans. To understand the role of the *Foxl2* gene in the gonad development of *P. clarkii*, we designed CDS-specific primers for the *P. clarkii Foxl2* (*PcFoxl2*) gene and cloned its CDS sequence using RT-PCR. The nucleotide and protein sequence information was then analyzed through bioinformatics analysis. The expression and subcellular localization of *PcFoxl2* in various tissues were detected using qRT-PCR and in situ hybridization. The effects of *PcFoxl2* knockdown on gonad development were investigated using RNA interference. The results showed that the CDS length of the *PcFoxl2* gene was 1614 bp and encoded 537 amino acids. Protein sequence comparison and phylogenetic analysis showed that *PcFoxl2* was the closest relative to Crayfish. qRT-PCR analysis indicated that the expression level of *PcFoxl2* in the testis was significantly higher (>40 fold) than that in the ovary (*p* < 0.01). The in situ hybridization results showed that *PcFoxl2* was expressed in both the cytoplasm and the nucleus of egg cells, and that the expression was strongest in egg cells at the early stage of yolk synthesis, while weak in the secondary oocytes. The positive signal was strongest in the spermatocyte nucleolus, while only a trace signal was observed in the cytoplasm. After interfering with the *PcFoxl2* gene using dsRNA, the expression of *PcFoxl2* in the RNA interference group was significantly lower than that in the control group, and this interference effect lasted for one week. Moreover, the gonad index of the experimental group was significantly lower than that of the control group (*p* < 0.05) after 10 days of *P. clarkii* cultivation following *PcFoxl2* knockdown. The expression levels of the *nanos* and *S3a* genes, which are related to gonad development, decreased significantly after *PcFoxl2* gene interference. The results suggest that the *Foxl2* gene is involved in the growth and development of gonads, particularly in the development of testis, and is related to the early development of oocytes. This study provides a theoretical basis for the artificial breeding of *P. clarkii*.

## 1. Introduction

*Procambarus clarkii* belongs to the genus *Procambarus*, family Cambaridae. It was introduced into Nanjing, Jiangsu, China, in 1929 [1], and has since gained popularity among consumers. It has been widely cultivated in various regions of China, and it has become the shrimp species with the fastest growing aquaculture area and production in China in recent years [2]. *P. clarkii* has the advantages of a short growth cycle, strong fecundation, high disease resistance, rich nutrition, and a delicious taste [3]. The main breeding methods for *P. clarkii* are pond and paddy field culture [4]. Although the breeding areas have increased year by year, over-fishing, a lack of experience in seedling rearing in the early years, and a mismatch between the market-supply capacity of germplasm and the development demand of aquaculture have become the major factors restricting the development of the *P. clarkii* industry [5]. Therefore, it is of great significance to explore and analyze the reproductive development and regulation mechanisms of *P. clarkii*. Currently, the underlying mechanisms of ovarian maturation in decapod crustaceans remain unclear [6]. As a significant freshwater aquaculture species in China, *P. clarkii*’s life cycle can be divided into five stages: fertilized egg; embryo development; sexual maturity; reproduction; and aging and death. The fertilized egg stage serves as the basis for its growth, and it is very important to improve the fertilization rate to ensure its industrial development [7]. Previous studies have confirmed that the hatching rate of laid eggs is much higher than that of non-laid eggs. Under natural conditions, the average hatching rate of laid eggs is only approximately 30% [8]. However, by artificially inducing synchronous laying through improving water quality, rational feeding and adjusting the water level the average hatching rate of laying eggs can be made to reach 80.0% [9]. However, standardized methods of gonadal development and exploration are still lacking. In order to promote its long-term development, molecular breeding research is gradually emerging. Studies have shown that forkheadboxL2 (*Foxl2*) is a transcription factor that plays a key role in animal sex differentiation and gonad development [10]. It is involved in resisting follicle apoptosis and maintaining ovarian reserve function [11], and is related to sex determination, premature ovarian failure, infertility, tumors, and small eyelid cleft syndrome [12]. In vertebrates, the loss of the *Foxl2* gene in mice results in the abnormal development of ovarian granulosa cells, resulting in follicular block and oocyte atresia [13]. *Foxl2* is also involved in the differentiation of granulosa cells in the ovaries of *Oryzias latipes* [14]. The expression of *Foxl2* is highest before gonadal differentiation in *Clarias fuscus*, but decreases significantly after gonadal differentiation [15]. *Foxl2* is a well-known gene involved in the sex differentiation of many species, and it plays a key role in the development and maintenance of ovarian function. When the gonad of *Monopterus albus* develops from ovary to testis, *Foxl2* transcripts are dramatically reduced [16]. Studies have revealed the continuous and specific expression of the *Foxl2* gene in follicular layer cells during the processes of ovarian differentiation, maturation, and maintenance in *Danio rerio* [17]. In *Huso huso*, *Foxl2* is mainly expressed in female gonads [18]. The *Foxl2* mRNA level in *Amur sturgeon* is considered a suitable marker for the identification of sex in its early developmental stages [19]. In the early stages of gonad development in *Scatophagus argus*, the expression of *Foxl2* in the ovaries is higher than that in the testis [20]. Among invertebrates, the highest *Foxl2* expression in *Crassostrea hongkongensis* is in the gonad, and it is involved in gonad development through the estrogen signaling pathway [21]. The expression of *Foxl2* was first observed in the cytoplasm of the ovarian reproductive and follicular cells of *Chlamys farreri* after the onset of ovarian differentiation [22]. Fan and collaborators discovered that *Foxl2* is an evolutionally conserved female sex gene that is specifically expressed in the ovaries of *Chlamys farreri* [23]. *Foxl2* is associated with gametogenesis in *Portunus trituberculatus* and is essential for gonadal differentiation and development [24]. *Foxl2* expression surges in the ovarian development and maturity stages of *Eriocheir sinensis*, negatively impacting the synthesis of vitelline proteinogen (vtg) [25]. Studies have cloned and identified the *Foxl2* gene in the genome of *Macrobrachium rosenbergii* via molecular cloning, bioinformatics analysis, in situ hybridization, and quantitative analysis. The *Foxl2* gene is highly expressed in the testis, vas deferens, and ovaries. During ovarian development, *Foxl2* expression is highest during stage I. *Foxl2* may play a role in gonadal development in both female and male *M. rosenbergii* [26]. Significant progress has been made in the study of *Foxl2* gene function in animals, but no relevant studies have been reported on the function and silencing effect of the *Foxl2* gene in *P. clarkii*. In this study, the full-length cDNA sequence of the *Foxl2* gene in *P. clarkii* was simulated, and its expression in various tissues and in ovarian tissues at different developmental stages was analyzed. Finally, the *Foxl2* gene was silenced using RNA interference technology to analyze the gonadal development of cultured shrimp after gene silencing. This study aims to explore the gonadal development mechanism of *P. clarkii* at the molecular level, providing a theoretical basis for the study of the regulatory mechanisms related to the gonadal development of *P. clarkii*.

## 2. Materials and Methods

### 2.1. Experimental Materials

In this study, healthy and evenly sized *P. clarkii* (length: 8.33 ± 0.30 cm, weight: 21.56 ± 0.12 g) were selected from Datang town, Nanning city, Guangxi province, China. After dissection, 9 tissue samples (heart, hepatopancreas, gill, ovary, testis, blood, intestine, brain, and muscle; each sample was mixed with the tissues of 3 shrimps) were placed in 2 mL EP tubes without enzymes and stored at −80 °C after adding Trizol (Invitrogen, Waltham, MA, USA). qPCR experiments were completed using the PrimeScript RT reagent kit with gDNA Eraser (Perfect Real Time) (TaKaRa Bio Inc., Dalian, China) and the real-time fluorescence quantitative reagent SYBR^®^ Premix Ex Taq™ II (Tli RnaseH Plus) (TaKaRa Bio Inc., Dalian, China). The interferons were synthesized using the HiScribe™ T7 Quick High Yield RNA Synthesis Kit (NEB, Ipswich, MA, USA).

### 2.2. Primer Design and Synthesis

Specific primers were designed based on the CDS sequence of the *Foxl2* gene obtained from the genome sequencing result of *P. clarkii* (NC_059599.1) published in GenBank, using the Primer Premier 5.0 software. The primers (Table 1) used in the experiment were all synthesized by Sangon Biotech company (Shanghai, China).

### 2.3. Total RNA Extraction and cDNA Synthesis

Total RNA was extracted from 9 tissues, including muscle, liver, and ovarian tissues, at different developmental stages using the Trizol method. The concentration and purity of the total RNA were detected using the NanoDrop 2000, and its integrity was detected using 1.5% agarose gel electrophoresis. The total RNA was reverse-transcribed to synthesize cDNA using the PrimeScript RT reagent kit with the gDNA Eraser (Perfect Real Time) kit following the manufacturer’s instructions. The reverse-transcribed samples were stored at −20 °C for later use.

### 2.4. PcFoxl2 Gene Cloning

In this study, the whole-tissue cDNA was mixed in equal volumes for use as a template (2.0 μL), and 1.0 μL each of *PcFoxl2* F and *PcFoxl2* R were added. PrimeSTAR Max DNA Polymerase (high-fidelity enzyme) was added at a volume of 5 μL, and dd H_2_O was added to give 20.0 μL. The amplification procedure was as follows: pre-denaturation was performed at 98 °C for 1 min; then, 35 cycles were performed at 98 °C for 10 s, 58 °C for 30 s, and 72 °C for 40 s. The amplification was extended for 5 min at 72 °C and terminated at 4 °C [26]. The PCR product was gel-purified and cloned into competent cell Top10 (TaKaRa, Osaka City, Osaka Prefecture, Japan) for sequencing.

### 2.5. Bioinformatics Analysis

The *PcFoxl2* nucleotide and amino acid sequences were analyzed using Sequence Manipulation Suite (SMS) (http://www.bio-soft.net/sms/, accessed on 1 August 2023). The molecular weight and theoretical isoelectric point were analyzed using ProtParam (https://web.expasy.org/protparam, accessed on 10 August 2023). The domain structure was analyzed using NCBI Conserved Domains (https://www.ncbi.nlm.nih.gov/cdd/, accessed on 16 August 2023) tools online. The amino acid sequence of *PcFoxl2* homology was searched using NCBI Blast, and a multi-sequence analysis of amino acids was performed using GeneDoc 2.7 software. Fifteen amino acid sequences of different species, including *Procambarus clarkii* (XP_045602999.1), *Cherax quadricarinatus* (XP_053640006.1), *Homarus americanus* (XP_042206756.1), *Penaeus vannamei* (XP_027218694.1), *Penaeus monodon* (XP_037795163.1), *Penaeus chinensis* (XP_047499877.1), *Eriocheir sinensis* (XP_050729799.1), *Scylla paramamosain* (QJQ31004.1), *Portunus trituberculatus* (XP_045109725.1), *Onychostoma macrolepis* (KAF4103853.1), *Danio rerio* (NP_001038717.1), *Peromyscus leucopus* (XP_037063755.1), *Onychomys torridus* (XP_036049215.1), *Mustela putorius furo* (XP_004762573.2), and *Homo sapiens* (NP_075555.1), were selected for construction of a phylogenetic tree using the Neighbor-Joining method in MEGA 11 software.

### 2.6. Expression of the PcFoxl2 Gene in Different Tissues of P. clarkii

A real-time PCR reaction was performed using the TB Green^®^ Premix Ex Taq™ II (Tli RNaseH Plus) kit. *18S rRNA* was used as the internal reference gene. The sequence of primers is shown in Table 1. The templates were cDNA from different tissues and ovarian tissues of *P. clarkii* at 5 developmental stages. The qRT-PCR reaction system was 20 μL, and the reaction procedure was divided into three steps: pre-denaturation at 95 °C for 30 s; 40 cycles of denaturation at 95 °C for 5 s; and renaturation at 60 °C for 34 s. The melting curve program was as follows: denaturation at 95 °C for 15 s; renaturation at 60 °C for 1 min; and denaturation at 95 °C for 15 s. The quantitative results were analyzed using the 2^−ΔΔCt^ calculation method. SPSS 27.0 and Microsoft Office Excel 2016 were used for the statistical analysis of the quantitative data, and one-way ANOVA and LSD were used to identify differences between the different data groups. *p* < 0.05 indicated a significant difference, and *p* < 0.01 indicated an extremely significant difference. Finally, GraphPad Prism 9 (GraphPad Software, San Diego, CA, USA) was used to construct the graphs.

### 2.7. In Situ Hybridization

Specific oligonucleotide probe sequences were designed using the Primer PremierX software based on the CDS sequence of the *PcFoxl2* gene, and the SweAMIFISH probes were synthesized by Servicebio (Wuhan, China) (Table 1). The lengths of the three probe primers were 28 bp, 27 bp, and 26 bp. The tissue samples of the ovary (3) and testis (3) of the paraffin-embedded *P. clarkii* were processed via tissue fixation, dehydration, sectionalization, digestion, pre-hybridization, hybridization, DAB color development, the restaining of the nucleus, dehydration sealing, etc. Finally, the in situ hybridization gonad sections under the positive and antisense probe were observed using a microscope and the images were collected and analyzed.

### 2.8. Exploration of the Function of PcFoxl2 in Gonadal Development

The cDNA of the purified CDS fragment of the *PcFoxl2* gene was amplified via PCR using the HiScribe™ T7 Quick High Yield RNA Synthesis Kit (NEB, Ipswich, MA, USA) interference kit, and the income of the dsRNA products was evaluated using agarose gel electrophoresis. We selected 24 healthy adult crayfish of uniform size for preliminary experiments, and these shrimp were divided into 4 groups, with 3 males and 3 females in each group. The *PcFoxl2* interference agent was injected into the shrimp via intramuscular injection at doses of 0 μg/g, 5 μg/g, 10 μg/g, and 15 μg/g, respectively, and diluted with PBS to 100 μL. After 24 h of interference, the gonadal samples of the shrimp were taken and analyzed via qRT-PCR to determine the optimal interference concentration. We selected 240 adult crayfish in similar condition for formal experiments, with a male/female ratio of approximately 1:1. These shrimp were divided into two groups, the ds-*Fox12* treatment group, and the ds-*GFP* control group, with 120 shrimp in each group. Three parallel experiments were conducted, with 40 shrimp in each group. The shrimp in the experimental group were injected with the *PcFoxl2* gene RNA interference reagent, and the shrimp in the control group were injected with the *GFP* gene RNA interference reagent. According to the results of the preliminary experiments, the optimal injection dose was 15 μg/g, diluted with PBS to 100 μL, and this was injected into the shrimp via intramuscular injection. The injection method for *GFP* in the control group was the same as above. The gonadal samples were taken at 24 h and analyzed via qRT-PCR to detect the expression of the *S3a* and *nanos* genes, which are related to gonadal development. Three shrimp were evaluated at 48, 96, 144, 168, 192, and 216 h via qRT-PCR to detect the effective time of interference. According to the preliminary experimental results, the effective time of *PcFoxl2* dsRNA interference was approximately one week, so continuous RNA interference was performed on the sixth day after the first injection. The samples were taken from the shrimp in each group on the 0th, 5th, 8th, and 10th days after injection, and after 10 days of culture, the gonadal indices of the male and female shrimp were measured via dissection.

Ovarian index (%) = ovarian mass/body mass of shrimp × 100

Testicular index (%) = testicular mass/body mass of shrimp × 100

## 3. Results

### 3.1. PcFoxl2 Gene Sequence Characteristics 

**Figure 1:**
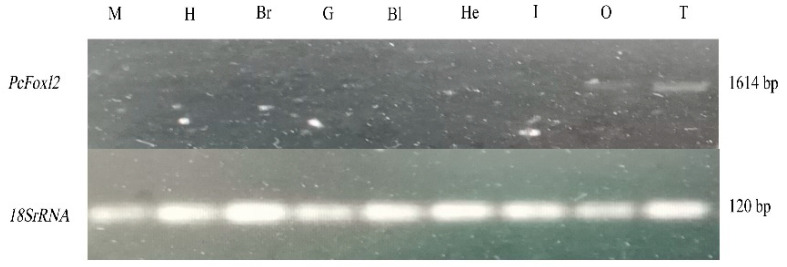
Agarose gel electrophoresis for PCR amplification products of the *Foxl2* gene in various tissues of *P. clarkii.* The letters above the figure represent: M, Muscle; H, Hepatopancreas; Br, Brain; G, Gill; B, Blood; H, Heart; I, Intestine; O, Ovary; T, Testis.

**Figure 2:**
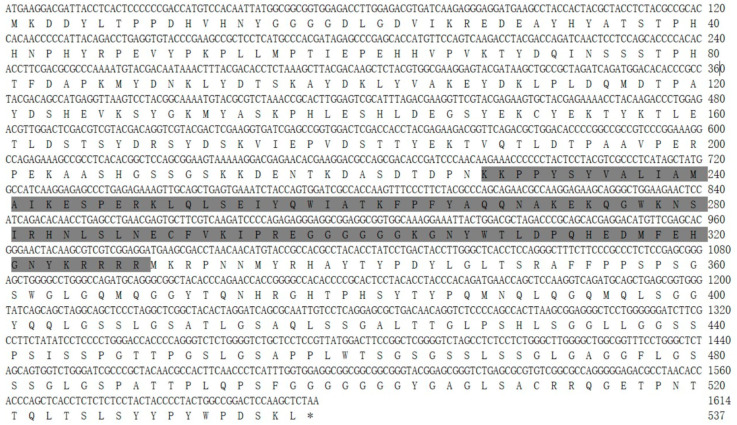
Amino acid sequence encoded by *Foxl2* gene in *P. clarkii.* The gray shaded area represents a forkhead (FH) domain.

The expression of the *PcFoxl2* gene in different tissues of *P. clarkii* was detected using an RT-PCR method. The results showed that the bands of the *PcFoxl2* gene PCR amplification products were only found in the ovary and testis tissues during electrophoresis, with the testis tissue showing the brightest bands and the highest expression level (Figure 1). The expression of *PcFoxl2* in ovarian tissue was the second highest, suggesting that *PcFoxl2* may be related to the gonadal development of *P. clarkii*. The genome sequencing results for *P. clarkii* (NC_059599.1) published by GenBank showed that the total length of the *PcFoxl2* gene is 5654 bp. However, according to the sequencing results and bioinformatics analysis of this study, the CDS sequence length of the *PcFoxl2* gene is 1614 bp, encoding 537 amino acids. The predicted molecular weight of the protein was 58.8 kDa, and the theoretical isoelectric point was 6.72. The K228-R328 region was a forkhead (FH) domain unique to the *Foxl2* gene family (Figure 2).

### 3.2. PcFoxl2 Amino Acid Homology Comparison and Phylogenetic Tree Construction

**Figure 3:**
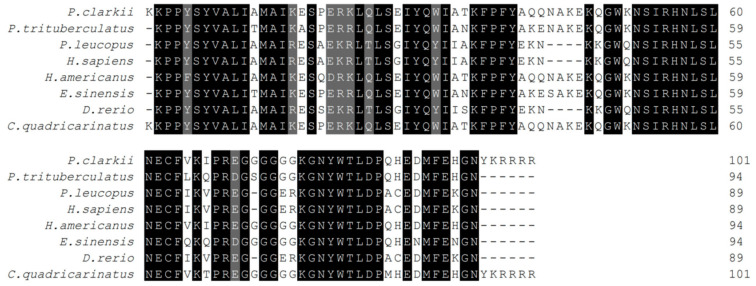
Multiple sequence alignment of an FH domain of *PcFoxl2* in *P. clarkii* and other species. The black areas represent highly similar domains, and the gray areas represent not very similar domains.

The results of the multiple amino acid comparison analysis showed that the *PcFoxl2* protein had a high similarity with the *Foxl2* protein of other species, and that the similarity with *Cherax quadricarinatus* was the highest (77.53%). The similarities with *Eriocheir sinensis* and *Portunus trituberculatus* (XP_045109725.1) were 59.86% and 61.06%, respectively. The similarity with *Danio rerio* was 17.67%. As for *Homo sapiens* (NP_075555.1) and *Peromyscus leucopus*, the similarities were 18.01% and 18.52%, respectively (Figure 3). These results indicate that the *Foxl2* gene sequence analysis demonstrated a high homology between *P. clarkii* and decapod *P. clarkii*.

**Figure 4:**
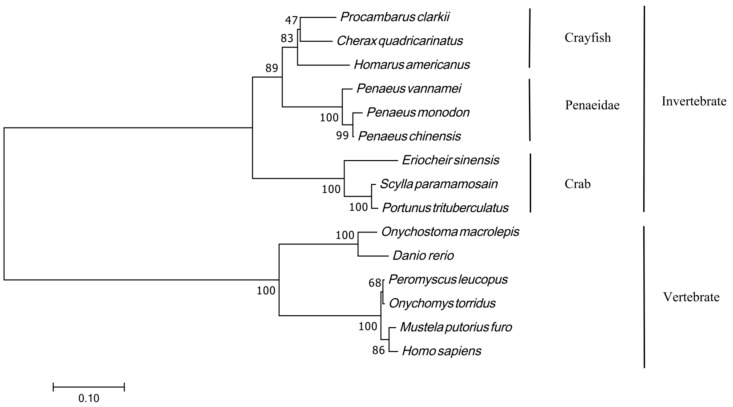
Neighbor-joining phylogenetic tree based on the *Foxl2* amino acid sequences. The following are the species names and IDs of the *Foxl2* amino acid sequences that were used to construct the phylogenetic tree: *Procambarus clarkii* (XP_045602999.1), *Cherax quadricarinatus* (XP_053640006.1), *Homarus americanus* (XP_042206756.1), *Penaeus vannamei* (XP_027218694.1), *Penaeus monodon* (XP_037795163.1), *Penaeus chinensis* (XP_047499877.1), *Eriocheir sinensis* (XP_050729799.1), *Scylla paramamosain* (QJQ31004.1), *Portunus trituberculatus* (XP_045109725.1), *Onychostoma macrolepis* (KAF4103853.1), *Danio rerio* (NP_001038717.1), *Peromyscus leucopus* (XP_037063755.1), *Onychomys torridus* (XP_036049215.1), *Mustela putorius furo* (XP_004762573.2), and *Homo sapiens* (NP_075555.1).

MEGA11.0 was used to construct a phylogenetic evolutionary tree using the maximum likelihood method, and the *Foxl2* gene protein sequences of shrimp, crabs, fish, and amphibians in different taxonomic positions were analyzed. The results showed that the phylogenetic tree was divided into two branches, and that invertebrates such as *P. clarkii*, *Homarus americanus*, and *Eriocheir sinensis* were grouped into one branch. Vertebrates such as *Homo sapiens*, *Peromyscus leucopus*, *Onychomys torridus* (XP_036049215.1), and *Danio rerio* were gathered in one group (Figure 4).

### 3.3. Expression Level of PcFoxl2 in the Gonadal Tissue

**Figure 5:**
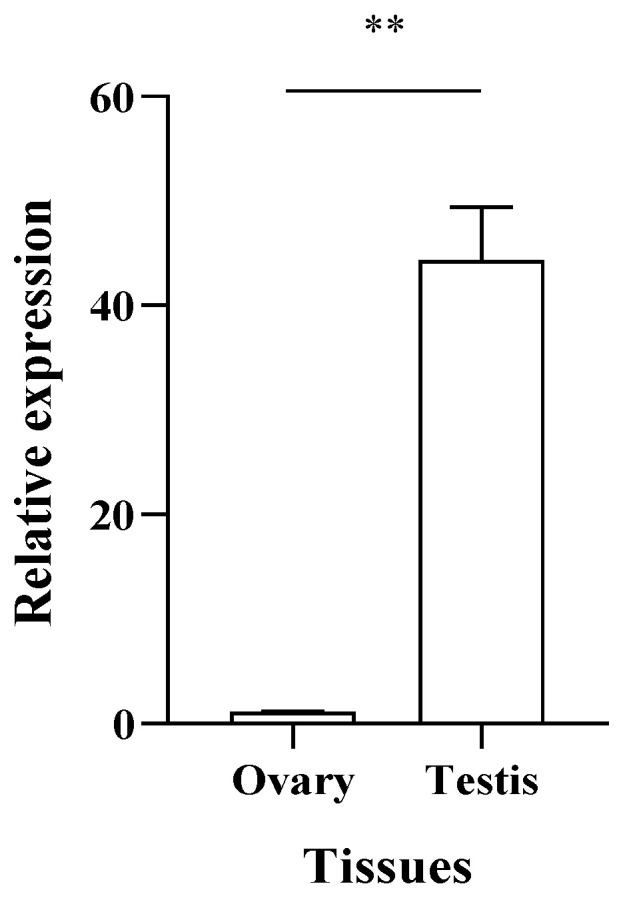
Relative expression level of *PcFoxl2* in the *P. clarkii* gonads. ** indicates extremely significant difference (*p* < 0.01).

Using *18SrRNA* as the internal reference gene, qRT-PCR was used to detect the expression level of the *PcFoxl2* gene in the gonadal tissues. The results showed that *PcFoxl2* was expressed in both the ovary and testis, and that the expression level in the testis was higher than that in the ovarian tissues (*p* < 0.01) (Figure 5). The expression level of *PcFoxl2* in the testis was 44 times higher than that in the ovaries, showing an obvious sex dimorphism.

### 3.4. Expression Pattern of PcFoxl2 during Ovarian Development

**Figure 6:**
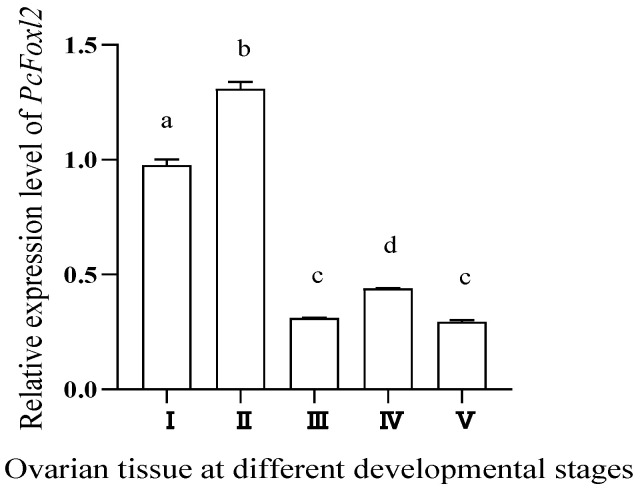
Expression of *PcFoxl2* in ovarian tissues at different stages. Different letters indicate significant differences (*p* < 0.05).

Using *18SrRNA* as the internal reference gene, the expression level of *PcFoxl2* was detected using qRT-PCR. The results showed that the expression level of the *PcFoxl2* gene was highest in ovarian stage II, and that the expression level in early ovarian development was significantly higher than that in late ovarian development (Figure 6), suggesting that this gene plays an important role in early oocyte development.

### 3.5. Subcellular Localization of PcFoxl2 in Gonadal Tissue

**Figure 7:**
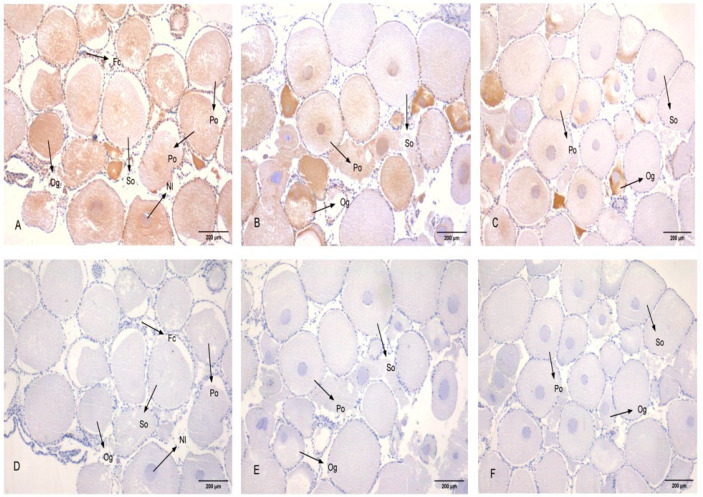
In situ hybridization of *Foxl2* mRNA in the ovary of *P. clarkii.* Yellow represents the positive signal and blue represents the nucleus. The figure shows follicular cells (Fc), oogonia (Og), primary oocytes (Po), secondary oocytes (So), and nucleoli (Nl). (**A**–**C**) show the results of in situ hybridization with the *PcFoxl2* mRNA antisense probe, and (**D**–**F**) show the results of in situ hybridization with the *PcFoxl2* mRNA sense probe.

**Figure 8:**
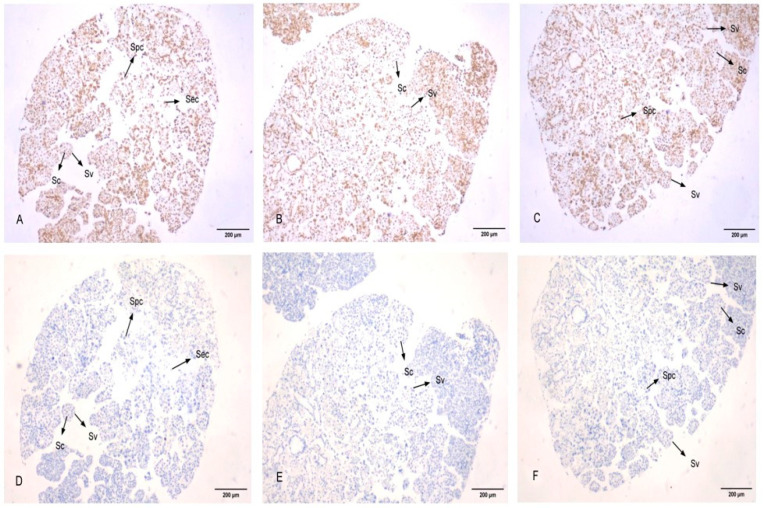
In situ hybridization of *Foxl2* mRNA in the testis of *P. clarkii.* Yellow represents the positive signal and blue represents the nucleus. The figure shows the Spermatogonia cell (Sc), spermatocytes cell (Spc), sperm cells (Sec), and seminal vesicles (Sv). (**A**–**C**) show the results of in situ hybridization with the *PcFoxl2* mRNA antisense probe, and (**D**–**F**) show the results of in situ hybridization with the *PcFoxl2* mRNA sense probe.

Experiments were carried out on the gonads of adult shrimp (three in each male and female). The germ cells of the *P. clarkii* include oogonia, primary oocytes, secondary oocytes, egg cells, follicular cells, spermatogonia, sperm cells, and so on [27]. The results revealed that *PcFoxl2* was expressed in the cytoplasm and nucleus of oocytes, primary oocytes, and secondary oocytes in the ovary of *P. clarkii*. Specifically, a strong positive signal was observed in oocytes and primary oocytes, a weaker signal in secondary oocytes, and no expression in follicular cells (Figure 7), which corresponds to the results of the qRT-PCR experiments. The egg cells of *P. clarkii* began to produce a large amount of yolk substances during the early stages of cell development [28], and *PcFoxl2* mRNA had a strong positive signal in the early egg cells of *P. clarkii*, suggesting that the *PcFoxl2* gene promotes the production of yolk substances in the ovary. In the testis, a large number of *PcFoxl2* mRNA positive signals were observed, which further confirmed that the expression level of *PcFoxl2* in the testis was significantly higher than that in the ovaries. Among them, the positive signal was the strongest in the sperm nucleolus, and only a trace signal was observed in the cytoplasm (Figure 8). This indicates that *PcFoxl2* is mainly expressed and plays a role in the sperm nucleoli. These results indicated that the *PcFoxl2* gene may play an important role in maintaining the normal function of oocytes, primary oocytes, and the testis.

### 3.6. Expression Effects of PcFoxl2 Gene after Interference

**Figure 9:**
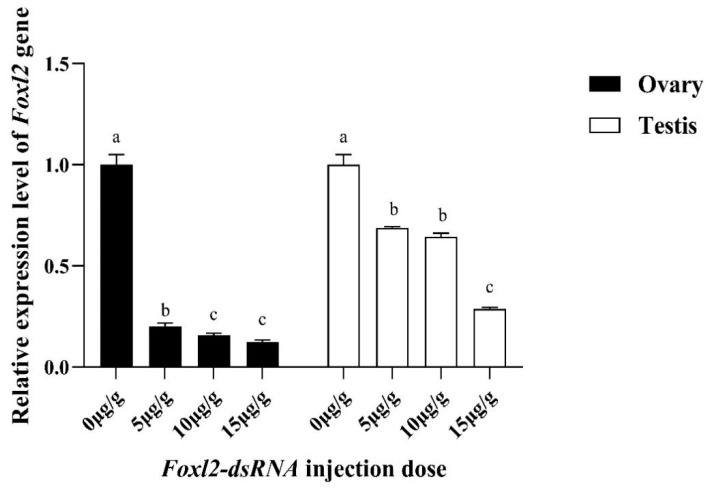
Relative expression of the *Foxl2* gene in *P. clarkii* under different dsRNA injection doses. Significantly different levels (*p* < 0.05) are denoted by different letters.

**Figure 10:**
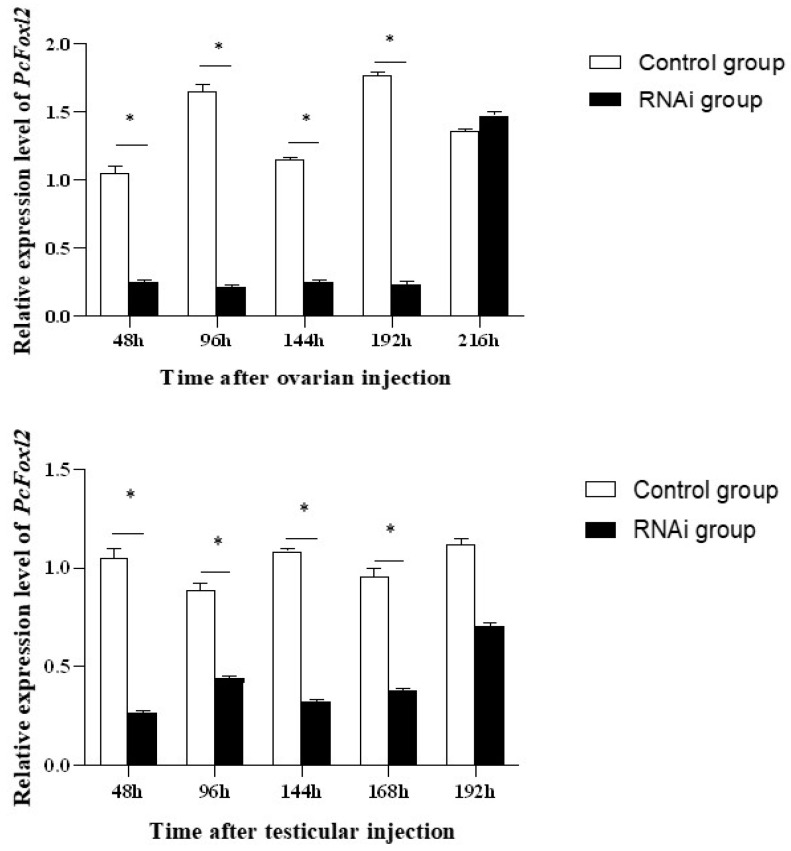
The effect duration of dsRAN interference on the *Foxl2* gene in *P. clarkii.* * indicates extremely significant difference (*p* < 0.05).

**Figure 11:**
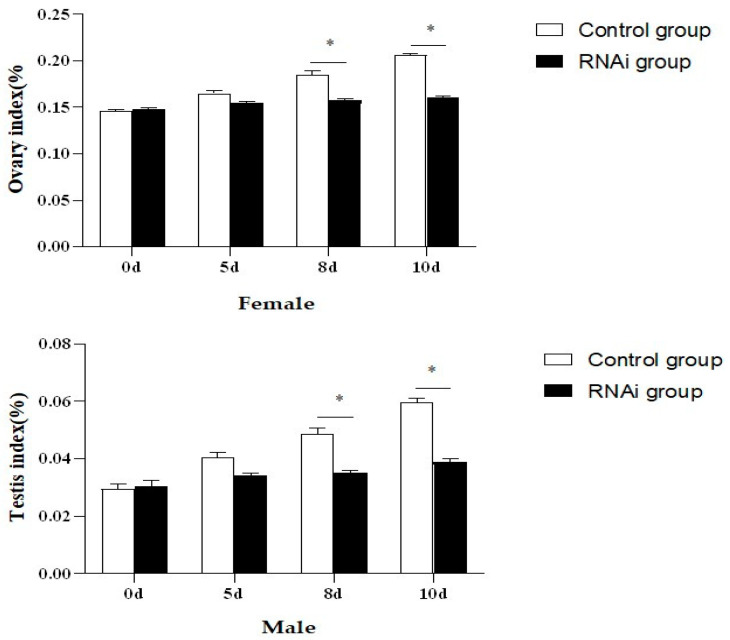
Ovarian index and testis index of *P. clarkii* at different times after *Foxl2* gene interference. * indicates extremely significant difference (*p* < 0.05).

In this study, the interference efficiency of *PcFoxl2* in the gonads was detected via qRT-RCR after the expression of *Foxl2* was reduced using RNAi technology. Three dsRNA injection doses of 5 μg/g, 10 μg/g, and 15 μg/g were tested in female and male shrimp, respectively. It was observed that the injection dose of 15 μg/g had the best knockdown effect on the *PcFoxl2* gene in both the ovary and testis, with an interference efficiency of approximately 78% in the ovary and 70% in the testis (Figure 9). The gonadal index of the experimental group and the control group was measured after ten days of breeding. The results showed that the gonadal index of the female shrimp in the interference group was 0.16 ± 0.015% and that of the male shrimp was 0.04 ± 0.002%. In comparison, the gonadal index of the female shrimp in the control group was 0.21 ± 0.023%, and that of the male shrimp was 0.06 ± 0.003% (Table 2). The expression of *Foxl2* in the female *P. clarkii* in the RNA interference group after 168 h of culture was significantly lower than that in the undisturbed group (Figure 10). On days eight and ten after continuous RNA interference, the gonad index of both male and female *P. clarkii* compared between the control and experimental groups showed significant differences, and that in the control group was significantly higher than that in the RNA interference group. Moreover, the gonad index showed an increasing trend (Figure 11).

### 3.7. Expression Characteristics of Genes Related to Gonadal Development in P. clarkii after PcFoxl2 Interference

**Figure 12:**
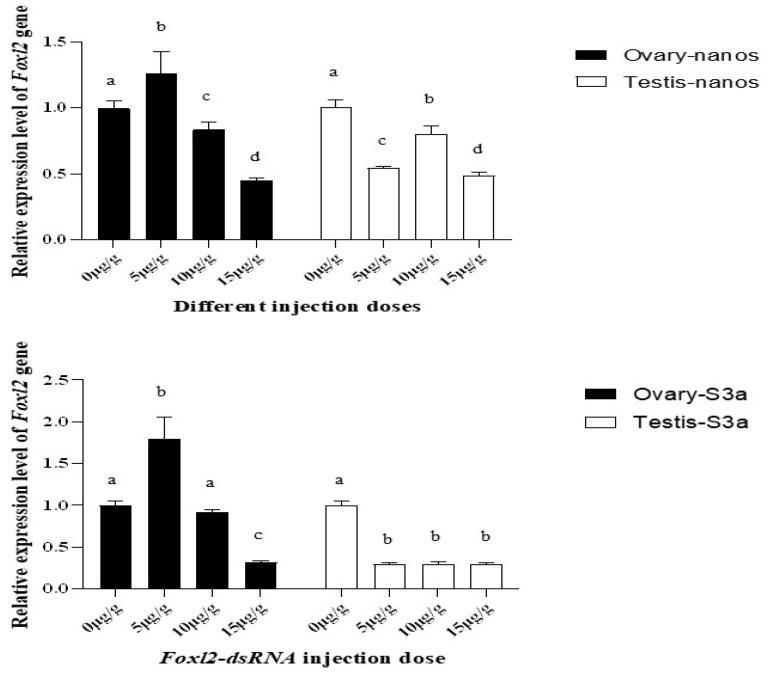
Changes in the expression level of *nanos* and *S3a* genes in the gonads of *P. clarkii* after *Foxl2* gene interference. Significantly different levels (*p* < 0.05) are denoted by different letters.

The above results showed that dsRNA at a dose of 15 μg/g had the best interference effect on *PcFoxl2*. After *PcFoxl2* gene interference, the expression level of the *nanos* gene, which is related to gonadal development, decreased to the lowest level in both the testis and ovaries when a dsRNA dose of 15 μg/g was applied; the decrease effect was also similar. This decrease was significantly lower than that at a dose of 0 μg/g (Figure 12). The black bar chart shows the expression of the *nanos* gene in the ovary, while the white bar chart shows the expression in the testis. Similarly, when the dose of dsRNA was 15 μg/g, the expression of the *S3a* gene in the ovary and testis decreased to the lowest level, and the expression of the *S3a* gene in the testis was also significantly affected at the injection doses of 5 μg/g and 10 μg/g (Figure 12). It was evident that the expression of the *S3a* gene in the testis was more easily regulated by the *PcFoxl2* gene.

## 4. Discussion

In this study, the *PcFoxl2* gene was cloned based on genomic data, and its expression pattern was revealed in various tissues of *P. clarkii*. The nucleotide and amino acid sequences of the gene were analyzed, and the subcellular localization of the gene in the gonads was determined via an in situ hybridization experiment. Furthermore, the *PcFoxl2* gene was silenced via the gene interference technique to explore its effect on gonad development. The results showed that the *PcFoxl2* CDS sequence was 1614bp long and encoded 537 amino acids, and it had the closest homologous relationship with *C. quadricarinatus* and *E. sinensis*. The *Foxl2* gene of *M. rosenbergii* was found to be most closely related to the *Foxl2* gene of *E. sinensis;* the cDNA of the *Foxl2* gene of *M. rosenbergii* encodes a protein with 489 amino acids and contains a highly conserved forkhead (FH) domain [26]. Similar to other *Foxl2* proteins, *PcFoxl2* contains a highly conserved forkhead (FH) domain, suggesting that *PcFoxl2* is conserved during evolution. The RT-PCR and qRT-PCR results showed that *PcFoxl2* was specifically expressed in the ovaries and testes, with an expression level in the testis that is significantly higher than that in the ovary (*p* < 0.01). The expression level of *PcFoxl2* in the testis is more than 44 times that in the ovary, revealing significant sexual dimorphism. Among the five stages of ovarian tissue, *PcFoxl2* is most highly expressed in stage II ovarian tissue. Studies have detected the partial sequence of the *Foxl2* gene in *P. clarkii* using transcriptome technology, with the gene being found to encode 339 amino acids and to be specifically expressed in the ovary, reaching its highest level during the yolk formation stage [29]. In this study, *PcFoxl2* was also specifically expressed in the ovary, reaching its highest level at the yolk development stage; this was consistent with previous studies. The *PcFoxl2* gene encodes 537 amino groups and is specifically expressed in the testis, with its expression level in the testis being more than 40 times that in the ovary. The expression level of *PcFoxl2* in female and male shrimp exhibited significant differences. It is speculated that *PcFoxl2* not only plays an important role in the regulation of early ovarian development, but also plays an important role in the males of *P. clarkii*. One study found that the length of the ORF region of *Foxl2* in *Hyriopsis cumingii* is 1215bp, encoding 404 amino acids, and that it also contains a conserved FH domain; however, the expression level of *Foxl2* in the ovary was significantly higher than that in the testis [30]. *Foxl2*, as an evolutionarily conserved gene, plays a key role in the early differentiation of animal gonads and the regulation of sex [31]. Among aquatic animals, studies have found that the *Foxl2* gene plays a key role in the sex differentiation of *Gnathopogon caerulescens* [32]. The *FoxL2* gene is specifically expressed in the ovary of *rainbow trout* [33], and it begins to be expressed in the ovary of *Nile tilapia* at the early stage of gonad differentiation; it continues to be expressed into adulthood, which means that it is involved in gonad differentiation and the maintenance of ovarian function [34]. The same results have been found in other fish. The *Foxl2* gene is mainly expressed in the brain, gill, and ovary of *Channa argus*, with the highest expression level being found in the ovary and a lower expression level being found in the testis and other tissues [35]. The *Foxl2* messenger RNA in *catfish* is expressed more significantly in the brain, pituitary gland, and ovary of females. However, its expression is low in the first two tissues and the testis of males [36]. Studies have found that the expression of the *Foxl2* gene is the highest in the gonads of female *Bettas plendens*, and that the expression of gonads in male fish is lower than that in female fish [37]. Some studies detected the *Foxl2* protein in the ovarian granulosa cells and testicular support cells of *Paralichthys olivaceus*, exhibiting a significant sex ditype expression pattern [38]. Unlike fish, in the tissues of *Macrobrachium Nipponense*, the expression of *Foxl2* in the testis is higher than that in the ovary; it is also higher in males than in females, suggesting that *Foxl2* may promote the process of sexual differentiation and development in *Macrobrachium nipponense* [39]. The results of the semi-quantitative PCR (Sq-PCR) tests revealed that the *Foxl2* gene of *Scylla paramamosain* was mainly expressed in the gonads (testis and ovary). The results of further real-time quantitative PCR (qRT-PCR) tests revealed that its expression level in the testis was significantly higher than that in the ovary (*p* < 0.01) [10]. In this study, the level of expression in the testis was significantly higher than that in the ovaries, which is similar to the results of other studies conducted on shrimp and crabs, and contrary to the results of studies conducted on fish. These findings revealed that the *Foxl2* gene exhibited evident sexual dimorphism in aquatic animals such as fish and shrimp, and that the expression level of the *Foxl2* gene in the ovaries of fish was much higher than that in the testis, which is contrary to the results of related studies conducted on shrimp and crabs. This indicates that the *Foxl2* gene is crucial for regulating the differentiation of sexual glands in aquatic animals. 

In situ hybridization experiments may be performed to confirm the specific expression location of detected genes in tissue cells and the strength of expression signals [40]. In this study, three male and three female *P. clarkii* gonads were selected for the observation of subcellular localization. The results revealed that *PcFoxl2* mRNA was strongly expressed in the cytoplasm and nucleus of oocytes and primary oocytes in the ovaries of female shrimp, and that positive hybridization signals were evenly distributed in the cytoplasm and nucleolus. Studies have demonstrated that a large volume of yolk is deposited in the cytoplasm of secondary oocytes [27], which indicates that the *PcFoxl2* gene primarily plays a regulatory role in the early stage of yolk formation, especially in the stage at which oocytes differentiate to primary oocytes. The *Foxl2* mRNA positive signal of Gulf Scallops was detected only in the ovaries, and there was no positive signal in follicular cells [41]. In this study, no positive signal was detected in follicular cells, and the positive signal was stronger in the testis, indicating that the levels of *Foxl2* mRNA in cells vary among invertebrates. The results of the in situ hybridization revealed that the *Foxl2* transcript was found in the spermatocyte, oocyte, and vasosecretory epithelial cells of *Macrobrachium rosenbergii* [26]. The in situ hybridization analysis found that the *Foxl2* signal was strong during the early development of the gonads in the *Macrobrachium nipponense* [39], which is similar to the results of this study. The expression characteristics of *Foxl2* in shrimp were similar.

In this study, for the first time, the function of the *Foxl2* gene in the gonadal development of *P. clarkii* was studied via RNAi experiments. First, three injection doses of 5 μg/g, 10 μg/g, and 15 μg/g dsRNA were administered to male and female shrimp, respectively, and tested. It was found that the injection dose of 15 μg/g had the optimum knockdown effect on the *PcFoxl2* gene. Long-term RNA interference may lead to significant changes in the phenotype of tissues or organs, and fewer injections is conducive to reducing the damage caused to the test animals [42]. It was found that the effect of the *PcFoxl2* gene on RNAi can be maintained for more than a week after the first injection of dsRNA. Because the interference experiment lasted for 10 days, the experimental animals were injected twice in total to maintain the interference effects. The RNA interference experiment on oysters revealed that feeding oysters with the dsRNA of *Foxl2* could minimize the expression of the *Foxl2* gene by 62–82%, and that the knockdown of *Foxl2* hindered the development of the gonads of female *Pacific oysters* [43]. In this study, the dsRNA of the *PcFoxl2* gene transcribed in vitro had an interference effect of approximately 78% in female shrimp and approximately 70% in male shrimp. According to the results of the qRT-PCR and in situ hybridization, the interference effect of the dsRNA injection was slightly lower than that in female shrimp because the expression of *Foxl2* was higher in male shrimp. The effect of *PcFoxl2* on the gonadal development of *P. clarkii* was explored by calculating the gonadal index using the gonadal tissue taken from *P. clarkii* for ten days after the knockdown of the *PcFoxl2* gene. The results showed that the gonadal index of the experimental group of *P. clarkii* and the male *P. clarkii* differed significantly from that of the control group after eight days of breeding; that is, the gonadal index of the shrimp after interference was significantly lower than that of the control group. The *PcFoxl2* gene exerts a regulatory effect on the development of the ovaries and testis of *P. clarkii*. After the interference of the *Foxl2* gene in *giant clam*, the length and diameter of oocytes did not increase significantly [44]. Studies have employed RNAi to knockdown *MrIR* in *Macrobrachium rosenbergii*, and one long-term knockdown experiment yielded neofemales. Via the performance of histological observations, it was inferred that an injection dose of 0.5 mu g/g body weight can efficiently retard spermatogenesis in the testes. The current study provides insights into MrIR knockdown-induced sex reversal in freshwater shrimp [42]. In this study, the gonad was weighed in order to analyze the effect of the *Foxl2* gene on gonad growth. This method has also been explored by our predecessors. Studies have used RNAi technology to interfere with the notch1 gene and have calculated the gonadal index after ten days of cultivation to determine that the notch1 gene affects the development of the ovaries in *Exopalaemon carinicauda* [45].

In crustaceans, genes such as *nanos* and *S3a* are believed to be closely related to gonad maturation [46,47,48]. Studies generally observe the changes in the expression levels of related genes to understand the coordination between *Foxl2* and the genes that are brought down. After silencing the *Foxl2* gene of *Macrobrachium rosenbergii*, the expression levels of the ovarian-related genes VG and EcR were observed. These led to speculation that *Foxl2* may play an important role in the process of ovarian maturation in *Macrobrachium rosenbergii* [49]. In *Epinephelus coioides*, *Foxl2* directly up-regulates the expression of the aromatase gene *cyp19a1a* to promote ovarian differentiation, while *Foxl3* directly inhibits the expression of the aromatase gene *cyp19a1a* and up-regulates the expression of *cyp11b* to promote testicular differentiation [50]. It was found in this study that, after *PcFoxl2* was silenced, the expression levels of the *nanos* and *S3a* genes in the ovary and testis were significantly reduced. Previous studies also found the *nanos* and *S3a* genes to be significantly expressed in the gonads [51,52]. In vitro experiments demonstrated that, compared with untreated ovarian explants, *S3a* significantly stimulated the expression of the ovarian hypertrophy protein (*SOP*) and translationally controlled tumor protein (*TCTP*) genes in shrimp, suggesting that *S3a* may stimulate ovarian development in *Fenneropenaeus merguiensis* [53]. It is evident that, when the *PcFoxl2* gene was silenced, the expression levels of *nanos* and *S3a* were significantly reduced. It is speculated that the *nanos* and *S3a* genes are regulated by the *PcFoxl2* gene and work together to affect the development of the gonads in *P. clarkii*.

## 5. Conclusions

In this study, the full-length CDS sequence of the *Foxl2* gene in *P. clarkii* was cloned for the first time. It was 1614 bp in length and encoded 537 amino acids. The protein sequence was most closely related to Langoustine, and its expression level in the testis was more than 40 times higher than that in the ovary (*p* < 0.01). The results of the in situ hybridization conducted showed that *PcFoxl2* expression was strongest in egg cells at the early stage of yolk synthesis, but weak in secondary oocytes. The positive signal was strongest in the spermatocyte nucleolus, and only a trace signal was found in the cytoplasm. After interfering with the *PcFoxl2* gene using dsRNA technology, the expression of *PcFoxl2* in the RNA interference group was significantly lower than that in the control group, and the interference effect lasted for one week. After interfering with the *Foxl2* gene of *P. clarkii*, the gonadal index of the experimental group was significantly lower than that of the control group after ten days of cultivation (*p* < 0.05). The expression levels of the *nanos* and *S3a* genes, which are related to gonad development, decreased significantly after *PcFoxl2* knockdown. These results indicate that the *Foxl2* gene is involved in the growth and development of the gonads of *P. clarkii*, which may be related to the early development of oocytes and the function of sex differentiation. This study provides a theoretical basis for the artificial breeding of *P. clarkii*.

## Figures and Tables

**Table 1:** Primer information for the *Foxl2* gene.

Primer	Sequence (5′–3′)	Application
Foxl2-F Foxl2-R	GGATCCATGAAGGACGATTACCTCACT CTCGAGGAGCTTGGAGTCCGGCCAGTA	RT-PCR
Foxl2-Pro	TTGTAGGTTTTCTCGTAGCACTTCTCGT	Probe of CISH
	CCTTCGTGTTCTCGTCCTTTTTACTTC	
	CCGTAGGACTTAACCTCATGGCTGTC	
Foxl2-QF Foxl2-QR	TGACTACCTTGGGCTCACCT TGCATCTGACCTTGGAGCTG	qPCR
18SrRNA-F 18SrRNA-R	AATGTCTCGTGGTGGAAAAG TTAACTGTTATCTTTACCTTCC	RT-PCR
18SrRNA-QF 18SrRNA-QR	GGGGAGGTAGTGACGAAAAAT TATACGCTAGTGGAGCTGGAA	qPCR
Foxl2-dsF Foxl2-dsR	AGTGGATCGCCACCAAGTTT CAGCGCTCCTGAGGACAAT	dsRNA
Foxl2-T7-dsF Foxl2-T7-dsR	GGATCCTAATACGACTCACTATAGGAGTGGATCGCCACCAAGTTT GGATCCTAATACGACTCACTATAGGCAGCGCTCCTGAGGACAAT	dsRNA

**Table 2:** Various indicators of *P. clarkii* after *PcFoxl2* interference.

Group	IW (g)	FW (g)	SR (%)	GSI (%)	
				Female	Male
Experimental group	20.94 ± 3.745	21.02 ± 3.744	50.833	0.16 ± 0.015	0.04 ± 0.002
Control group	20.99 ± 4.257	21.08 ± 4.252	55.833	0.21 ± 0.023	0.06 ± 0.003

IW—Initial weight, FW—Final weight, SR—Survival rate, GSI—gonadosomatic indices.

## Data Availability

All data were downloaded from NCBI.
